# Ovarian cysts in women receiving tamoxifen for breast cancer

**DOI:** 10.1038/sj.bjc.6690280

**Published:** 1999-04

**Authors:** M J E Mourits, E G E de Vries, P H B Willemse, K A ten Hoor, H Hollema, W J Sluiter, H W A de Bruijn

**Affiliations:** Departments of Obstetrics and GynaecologyMedical OncologyPathology andEndocrinology, University Hospital Groningen, Hanzeplein 1, GZ Groningen, 9713, The Netherlands

**Keywords:** ovarian cysts, tamoxifen, breast cancer, chemotherapy, oestradiol, FSH

## Abstract

Tamoxifen is a nonsteroidal anti-oestrogen with gynaecological side-effects. Only recently, ovarian cyst formation during tamoxifen treatment has been reported. The present study aimed to evaluate patient-related parameters that determine ovarian cyst formation in women using tamoxifen for breast cancer. A cross-sectional study was performed in 142 breast cancer patients using tamoxifen. Forty-five patients were also examined prior to tamoxifen treatment. Gynaecological assessment, transvaginal ultrasonography (TVU) and serum oestradiol (E_2_) and follicle stimulating hormone (FSH) analysis were performed. Follow-up assessments were performed twice a year. Uni- or bilateral ovarian cysts were detected by TVU in 24 tamoxifen-using patients and in one patient before tamoxifen treatment. Multiple regression analysis showed that cyst development is related (multiple *R* = 0.73) to high E_2_ (*P* < 0.001), younger age (*P* < 0.001) and absence of high-dose chemotherapy (*P* = 0.007). Patients with ovarian cysts had higher serum E_2_ levels compared to patients without cysts (1.95 vs 0.05 nmol l^−1^; *P* < 0.001). All patients after high-dose chemotherapy or older than 50 years had E_2_ < 0.10 nmol l^−1^ and/or amenorrhoea > 1 year and did not develop ovarian cysts. Patients still having a menstrual cycle during tamoxifen had a high chance (81%) of developing ovarian cysts. Breast cancer patients receiving tamoxifen only develop ovarian cysts if their ovaries are able to respond to FSH stimulation as shown by E_2_ production. © 1999 Cancer Research Campaign


*British Journal of Cancer* (1999) **79**(11/12), 1761–1764
© 1999 Cancer Research Campaign
Article no. bjoc.1998.0280

### Ovarian cysts in women receiving tamoxifen for breast cancer

**MJE Mourits[1], EGE de Vries[2], PHB Willemse[2], KA ten Hoor[1], H Hollema[3], WJ Sluiter[4], HWA de Bruijn[1] and AGJ van der Zee[1]**

Departments of [1]Obstetrics and Gynaecology, [2]Medical Oncology, [3]Pathology and [4]Endocrinology, University Hospital Groningen, Hanzeplein 1, 9713 GZ Groningen, The Netherlands

**Summary** Tamoxifen is a nonsteroidal anti-oestrogen with gynaecological side-effects. Only recently, ovarian cyst formation during tamoxifen treatment has been reported. The present study aimed to evaluate patient-related parameters that determine ovarian cyst formation in women using tamoxifen for breast cancer. A cross-sectional study was performed in 142 breast cancer patients using tamoxifen. Forty-five patients were also examined prior to tamoxifen treatment. Gynaecological assessment, transvaginal ultrasonography (TVU) and serum oestradiol ($E_2$) and follicle stimulating hormone (FSH) analysis were performed. Follow-up assessments were performed twice a year. Uni- or bilateral ovarian cysts were detected by TVU in 24 tamoxifen-using patients and in one patient before tamoxifen treatment. Multiple regression analysis showed that cyst development is related (multiple $R = 0.73$) to high $E_2$ ($P < 0.001$), younger age ($P < 0.001$) and absence of high-dose chemotherapy ($P = 0.007$). Patients with ovarian cysts had higher serum $E_2$ levels compared to patients without cysts (1.95 vs 0.05 nmol $l^{-1}$; $P < 0.001$). All patients after high-dose chemotherapy or older than 50 years had $E_2 < 0.10$ nmol $l^{-1}$ and/or amenorrhoea > 1 year and did not develop ovarian cysts. Patients still having a menstrual cycle during tamoxifen had a high chance (81%) of developing ovarian cysts. Breast cancer patients receiving tamoxifen only develop ovarian cysts if their ovaries are able to respond to FSH stimulation as shown by $E_2$ production.

**Keywords:** ovarian cysts; tamoxifen; breast cancer; chemotherapy; oestradiol; FSH

Tamoxifen is a nonsteroidal anti-oestrogen that is widely prescribed to breast cancer patients either as palliative therapy in metastatic disease or as adjuvant therapy. In current trials in the UK, USA and Italy, the value of tamoxifen for the prevention of breast cancer in high-risk women is also investigated (Powles et al, 1994; Fisher and Redmond, 1991). Over the last decade concern about the side-effects of tamoxifen has focused mainly on the occurrence of endometrial carcinoma with a 2.3–6.4-fold increased risk compared to non-using patients (Fornander et al, 1989; Andersson et al, 1991; Van Leeuwen et al, 1994). Ovarian cyst formation during tamoxifen has been reported in single cases (Jolles et al, 1990; Barbieri et al, 1993; Seoud et al, 1993; Terada et al, 1993; Cohen et al, 1994; Shulman et al, 1994; Re et al, 1994), in a breast cancer prevention study (Powles et al, 1994) and in series of tamoxifen-treated breast cancer patients (Boccardo et al, 1992; Cohen et al, 1996; Shushan et al, 1996). However, it is unclear which patients are prone to develop ovarian cysts and if the presence of cysts is of any clinical relevance.

The present study was undertaken to evaluate parameters determining ovarian cyst formation. Patient characteristics like age, last menstrual period, previous chemotherapy, duration of tamoxifen use and serum $E_2$ and FSH levels were related to the occurrence of ovarian cysts.

*Received 3 March 1998*
*Revised 12 September 1998*
*Accepted 14 October 1998*

*Correspondence to:* MJE Mourits

### MATERIALS AND METHODS

A cross-sectional study was performed in pre- and post-menopausal breast cancer patients using tamoxifen. Patients from the Departments of Medical Oncology and Radiotherapy of the University Hospital in Groningen, the Netherlands were referred during tamoxifen treatment to the Department of Gynaecology, as part of a gynaecological screening programme for women using tamoxifen. Based on previous chemotherapy, three different patient groups were defined. Patients in groups A and B participated in a randomized trial comparing the efficacy of intensive adjuvant chemotherapy: group A had five cycles of standard dose FEC (5-fluorouracil, epirubicin and cyclophosphamide) and group B had four cycles of standard dose FEC and one cycle of high-dose chemotherapy (cyclophosphamide, thiotepa and carboplatin), followed by reinfusion of peripheral haematopoietic stem cells. In both groups, chemotherapy was followed by 2 years of tamoxifen. In group C, patients with advanced breast cancer were treated with tamoxifen without previous chemotherapy. Forty-five patients also underwent a gynaecological assessment, TVU and serum sampling prior to starting tamoxifen treatment. After the first visit all patients were seen for follow-up twice a year.

A gynaecological history was taken with emphasis on menstrual pattern and last menstrual period. After pelvic examination, all patients underwent transvaginal ultrasonography (TVU) using an Aloka 620 with a 5.0 MHz transvaginal transducer (Aloka, Tokyo, Japan). The same physician performed all examinations. Both ovaries were scanned and measured, counting and measuring individual ovarian cysts in three directions. Cysts were defined as sonolucencies in the ovary with a mean diameter of ≥ 30 mm (Granberg et al, 1989). Uterine size and endometrial thickness

**Table 1** Patient characteristics

| | All patients (*n* = 142) | Group A (*n* = 64) | Group B (*n* = 50) | Group C (*n* = 28) |
|---|---|---|---|---|
| Age in years median (range) | 48 (24–86) | 45 (27–56) | 46 (24–54) | 60 (44–86) |
| Previous chemotherapy | | | | |
| 5 × FEC | 64 | 64 | – | – |
| 4 × FEC + high-dose | 50 | – | 50 | – |
| No chemotherapy | 28 | – | – | 28 |
| Tamoxifen dose | | | | |
| 20 mg day$^{-1}$ | 25 | 4 | 1 | 20 |
| 40 mg day$^{-1}$ | 117 | 60 | 49 | 8 |
| Duration of amenorrhoea | | | | |
| ≤ 1 year | 67 | 41 | 22 | 4 |
| > 1 year | 75 | 23 | 28 | 24 |
| Ovarian cysts | | | | |
| Yes | 24 | 20 | 1 | 3 |
| No | 118 | 44 | 49 | 25 |

**Table 2** Ovarian cyst formation in tamoxifen users in relation to patient characteristics

| | No cysts | Cysts | *P* value |
|---|---|---|---|
| Number of patients | 118 | 24 | |
| Age in years (range) | 50 (24–86) | 38 (27–50) | < 0.05 |
| Amenorrhoea duration in months (range) | 24 (1–430) | 1 (1–11) | < 0.001 |
| Number of patients with amenorrhoea ≤ 1 yr | 43 | 25 | < 0.001 |
| Tamoxifen use in months (range) | 7 (0–92) | 5 (0–79) | N.S. |
| $E_2$ nmol l$^{-1}$ (range) | 0.05 (0–1.27) | 1.95 (0.17–4.8) | < 0.001 |
| FSH IU l$^{-1}$ (range) | 22.5 (7.8–51.2) | 12.8 (3.1–44.4) | N.S. |

were recorded and will be reported elsewhere. During each visit, serum samples were obtained. Serum FSH levels were determined by radioimmunoassay (Amerlite FSH Assay, Johnson & Johnson Clinical Diagnostics Ltd, Amersham, UK). For $E_2$ measurement an in-house radioimmunoassay was used as described previously (Jurjens et al, 1975). Normal values in premenopause for FSH are 0.5–10.0 IU l$^{-1}$ and for $E_2$ are 0.07–1.00 nmol l$^{-1}$, excluding midcycle peaks. Normal postmenopausal values are for FSH > 30 IU l$^{-1}$ and for $E_2$ < 0.10 nmol 1$^{-1}$ (Speroff et al, 1994).

### Statistics

Statistical analysis of the distribution of clinical and hormonal parameters in the two patient groups was performed with the Mann-Whitney *U*-test for unpaired samples. Frequency tables were analysed using the Fisher's exact or Chi-square test with Yates' correction for small numbers. Rank order correlations were calculated by the method of Spearman. To determine the importance of different patient parameters for ovarian cyst formation multiple regression analysis was performed. Only *P* values < 0.05 were considered significant.

### RESULTS

### Patient characteristics

Between January 1995 and October 1997, 142 consecutive breast cancer patients were referred to the gynaecology outpatient clinic. Patient characteristics at referral are summarized in Table 1. One hundred and seventeen patients (82%) received tamoxifen in a daily dose of 40 mg and 111/142 (78%) received previous chemotherapy. Forty-five patients (32%) were also examined before starting tamoxifen treatment.

No cyst-related complaints were recorded. Uni- or bilateral ovarian cysts were detected by TVU in 25 patients which could be palpated in 16 patients. Patient characteristics in relation to ovarian cyst formation are summarized in Table 2. Of the 25 cystic ovaries, 24 were detected in 142 patients using tamoxifen (17%), and one in 45 patients before tamoxifen treatment was started (2.2%) (*P* < 0.001). The mean diameter of the ovarian cysts varied from 31 mm to 61 mm and all had a benign aspect according to the criteria described by Granberg et al (1989). During follow-up the cysts either increased (32%) or decreased (36%) in size or they disappeared completely (32%). During the study, new cysts developed in the contralateral ovary in 41%. No medical or surgical intervention was indicated in any patient.

All cystic ovaries appeared in patients with a last menstrual period within 1 year (24/67 (36%)) and no cysts were found in patients with an amenorrhoea > 1 year (0/75 (0%)). Sixteen out of 142 tamoxifen-using patients not only had their last period less than 1 year ago but also maintained menstrual cycles during tamoxifen treatment. Thirteen (81%) of these patients developed ovarian cysts. All patients who had received high-dose chemotherapy became amenorrhoeic and no ovarian cysts developed in this group. Patients with ovarian cysts were younger (*P* < 0.05) and had a shorter period of amenorrhoea (*P* < 0.001) (Table 2). No cysts occurred in patients > 50 years. No relation was observed between dose or duration of tamoxifen use and the appearance of ovarian cysts.

Patients with cysts had higher serum $E_2$ levels (1.95 nmol l$^{-1}$) than patients without cysts (0.05 nmol l$^{-1}$; *P* < 0.001). Multiple regression analysis showed that ovarian cyst formation is related

 © Cancer Research Campaign 1999

(multiple $R = 0.73$) to high $E_2$ levels ($P < 0.001$), no previous high-dose chemotherapy ($P = 0.007$), and younger age (< 50 years; $P < 0.001$). These three parameters are associated, however.

In the 45 patients who were examined before the start of tamoxifen treatment, one ovarian cyst was detected in a 33-year-old patient who had received five cycles of standard dose FEC. After the patient started with tamoxifen the ovarian cyst first increased, later decreased in size and a new cyst developed in the contralateral ovary. After 2 years of tamoxifen treatment both cysts disappeared spontaneously.

### DISCUSSION

Tamoxifen is a nonsteroidal anti-oestrogen which has proven to be effective in the treatment of early and metastatic breast cancer (Early Breast Cancer Trialists' Collaborative Group, 1992; Santen et al, 1990). The present study shows that in patients with residual ovarian function, as defined by $E_2$ levels $\geq 0.10$ nmol $l^{-1}$, tamoxifen can result in ovarian cyst formation. Ovarian cysts appeared in 36% of tamoxifen-using patients with amenorrhoea of $\leq 1$ year. Apparently, ovarian cysts occurred only in functional ovaries as 13/16 patients (81%) with menstrual periods during tamoxifen treatment developed ovarian cysts, against no patients with amenorrhoea of more than 1 year. All patients who had received high-dose chemotherapy developed amenorrhoea and low $E_2$ (< 0.10 nmol $l^{-1}$) while no ovarian cysts were found in these patients. The results of the randomized trial comparing the efficacy of adjuvant standard-dose and high-dose chemotherapy in women $\leq$ 55 years of age are not yet available. Our findings of complete and permanent amenorrhoea in premenopausal patients treated with high-dose regimens might become one of the possible factors involved, if a difference in clinical outcome would be observed (Bines et al, 1996).

In a recent study by Cohen et al (1996), 16 out of 175 postmenopausal breast cancer patients treated with tamoxifen underwent salpingo-oophorectomy for various reasons. In 10 out of these 16 postmenopausal patients, ovarian enlargement was found during surgery. TVU prior to surgery detected ovarian cysts in seven. Pathologic examination showed benign cysts, cystadenomas, metastatic breast cancer in two and endometroid adenocarcinoma in one. No serum hormone levels were reported in this study. A direct relation between the variety of ovarian pathology and previous tamoxifen in this study is debatable. In the present study, no cysts were detected in postmenopausal patients. In a placebo controlled tamoxifen chemoprevention trial in 1054 healthy pre- and postmenopausal women (Powles et al, 1994) TVU demonstrated a significantly increased risk of ovarian cysts in premenopausal women who had received tamoxifen for more than 3 months. No change was found in ovarian appearances in postmenopausal women.

In the first GROCTA (Breast Cancer Adjuvant Chemo-hormone Therapy Cooperative Group) trial, three ovarian cysts were detected in 79 premenopausal patients on tamoxifen and no cysts in postmenopausal women or patients treated with chemotherapy ($n = 165$) or a combination of both treatments ($n = 171$) (Boccardo et al, 1992). Neither in the Royal Marsden Study nor in the GROCTA trial were data provided on hormonal status.

Our study shows that the effects of tamoxifen in premenopausal women differ from those observed in postmenopausal women. In premenopausal women tamoxifen use can result in ovarian cysts and possibly multiple ovulations. Data from both epidemiological and in vitro studies point to a link between incessant ovulation and malignant ovarian transformation (Wittemore et al, 1992). The long-term (> 1 year) use of clomiphene, another anti-oestrogen with poly-ovulatory effects on the ovaries (Gerhard and Runnebaum, 1979), has been linked to a higher risk of malignant ovarian tumours (Rossing et al, 1994). Current cancer prevention trials are predominantly performed in premenopausal women who are at increased risk of developing breast cancer. Some of these women will be carriers of a mutated BRCA1 or BRCA2 gene and are therefore also at risk of developing ovarian cancer (Shattuck-Eidens et al, 1995). Recently, a protective effect of oral contraceptive agents against ovarian cancer was recognized in BRCA1 and BRCA2 gene mutation carriers (Narod et al, 1998), most probably due to the inhibition of ovulation. Hypothetically, ovarian hyperstimulation by tamoxifen might have the opposite effect. Although tamoxifen has been prescribed to many premenopausal women since its approval, less premenopausal than postmenopausal patients have been treated with tamoxifen and the fact that no increased incidence of ovarian cancer has been reported thus far is therefore not completely reassuring (Spicer et al, 1991). In carriers of a mutated BRCA1 or BRCA2 gene ovarian enlargement may present a dilemma to the clinician.

In the present study, the ovarian cysts were all asymptomatic and had a benign ultrasonographic aspect. Follow-up of the cysts showed that they disappeared in time or (in one patient) after discontinuation of tamoxifen. Therefore, no medical or surgical intervention was indicated in our study population. However, complications of ovarian cysts such as ovarian torsion (Barbieri et al, 1993) and necrosis (Jolles et al, 1990) have been described in tamoxifen users. As the indications for tamoxifen use have increased and include preventative therapy for high-risk women, many healthy premenopausal women will be using tamoxifen in the near future.

This study shows that cysts in tamoxifen-using breast cancer patients develop only if their ovaries are able to respond to tamoxifen as indicated by $E_2$ production. Patients with menstrual periods during tamoxifen treatment have a high chance of developing ovarian cysts. We did not find any clinical consequences of the development of ovarian cysts in premenopausal women treated with tamoxifen in this study. However, awareness is warranted in those premenopausal women who are at increased risk of ovarian cancer because of BRCA1 or BRCA2 gene mutation.

### REFERENCES

Andersson M, Storm HH and Mouridson HT (1991) Incidence of new primary cancers after adjuvant tamoxifen therapy and radiotherapy for early breast cancer. *J Natl Cancer Inst* **83**: 1013–1017

Barbieri RL, Ferracci AL, Droesch JN and Rochelson BL (1993) Ovarian torsion in a premenopausal woman treated for breast cancer. *Fertil Steril* **59**: 459–460

Bines J, Oleske DM and Cobleigh MA (1996) Ovarian function in premenopausal women treated with adjuvant chemotherapy for breast cancer. *J Clin Oncol* **14**: 1718–1729

Boccardo F, Rubagotti A, Amoroso D, Sismondi P, Genta F, Nenci I, and other participants in the GROCTA (1992) Chemotherapy versus tamoxifen versus chemotherapy plus tamoxifen in node-positive oestrogen-receptor positive breast cancer patients. An update at 7 years of the 1st Grocta (Breast Cancer Adjuvant Chemo-hormone Therapy Cooperative Group) Trial. *Eur J Cancer* **28**: 673–680

Cohen I, Beyth Y, Tepper R, Shapira J, Zalel Y, Figer A, Cordoba M, Yigael D and Altaras MM (1996) Ovarian tumors in postmenopausal breast cancer patients treated with tamoxifen. *Gynecol Oncol* **60**: 54–58

Cohen I, Rosen DJD, Altaras MM, Beyth Y, Shapira J and Yigael D (1994) Tamoxifen treatment in premenopausal breast cancer patients may be associated with ovarian over-stimulation, cystic formations and fibroid overgrowth. *Br J Cancer* **63**: 620–621

© Cancer Research Campaign 1999

Early Breast Cancer Trialists' Collaborative Group (1992) Systemic treatment of early breast cancer by hormonal, cytotoxic or immune therapy. *Lancet* **339**: 1–15

Fisher B and Redmond C (1991) New perspective on cancer of the contralateral breast: a marker for assessing tamoxifen as a preventive agent. *J Natl Cancer Inst* **83**: 1278–1280

Fornander T, Cedermark B, Mattson A, Skoog L, Theve T, Askergren J, Rutquist LE, Glas U, Silfversward C, Somell A, Wilkins N and Hjalmar ML (1989) Adjuvant tamoxifen in early breast cancer: occurrence of new primary cancers. *Lancet* **i**: 117–120

Gerhard I and Runnebaum B (1979) Comparison between tamoxifen and clomiphene therapy in women with anovulation. *Archives of Gynaecology* **227**: 279–288

Granberg S, Wikland M and Jansson I (1989) Macroscopic characterization of ovarian tumors and the relation to the histological diagnosis: criteria to be used for ultrasound evaluation. *Gynecol Oncol* **35**: 139–144

Jolles CJ, Smotkin D, Ford KL and Jones KP (1990) Cystic ovarian necrosis complicating tamoxifen therapy for breast cancer in a premenopausal woman: a case report. *J Reprod Med* **35**: 299–300

Jurjens H, Pratt JJ and Woldring MG (1975) Radioimmunoassay of plasma estradiol without extraction and chromatography. *J Clin Endocrinol Metab* **40**: 35–41

Narod SA, Risch H, Moslehi R, Dorum A, Neuhausen S, Olsson H, Provencher D, Radice P, Evans G, Bishop S, Brunet J-S and Ponder BAJ (1998) Oral contraceptives and the risk of hereditary ovarian cancer. *N Engl J Med* **339**: 424–428

Powles TJ, Jones AL, Ashley SE, O'Brien MER, Tidy VA, Treleaven J, Cosgrove D, Nash AG, Sacks N, Baum M, McKinna JA and Davey JB (1994) The Royal Marsden Hospital pilot tamoxifen chemoprevention trial. *Breast Cancer Res Treat* **31**: 73–82

Re A, Wierdis T, Tassarolo M, Leo L, Bellino R, Lauricella A and Lanza A (1994) Two cases of ovarian cysts in postmenopausal patients under antiestrogen treatment. *Clin Exp Obstet Gynecol* **4**: 221–224

Rossing MA, Daling JR, Weiss NS, Moore DE and Self SG (1994) Ovarian tumors in a cohort of infertile women. *N Engl J Med* **331**: 771–776

Santen RJ, Manni A, Harvey H and Redmond C (1990) Endocrine treatment of breast cancer in women. *Endocr Rev* **11**: 221–265

Seoud MAF, Johnson J and Weed JC (1993) Gynecologic tumors in tamoxifen-treated women with breast cancer. *Obstet Gynecol* **82**: 165–169

Shattuck-Eidens D, McClure M, Simard J, Labrie F, Narod S, Couch F, et al (1995) A collaborative survey of 80 mutations in the BRCA1 breast and ovarian cancer susceptibility gene. Implications for presymptomatic testing and screening. *JAMA* **273**: 535–541

Shulman A, Cohen I, Altaras MM, Maymon R, Ben-Nun I, Tepper R and Beyth Y (1994) Ovarian cyst formation in two premenopausal patients treated with tamoxifen for breast cancer. *Hum Reprod* **9**: 1427–1429

Shushan A, Peretz T, Uziely B, Lewin A and Mor-Yosef S (1996) Ovarian cysts in premenopausal and postmenopausal tamoxifen-treated women with breast cancer. *Am J Obstet Gynecol* **174**: 141–144

Speroff L, Glass RH and Kase NG (1994) Regulation of the menstrual cycle. In: *Clinical gynecologic endocrinology and infertility*, Mitchell (ed), 5th edn, pp 183–230. Williams and Wilkins: Baltimore

Spicer DV, Pike MC and Henderson BE (1991) Ovarian cancer and long-term tamoxifen in premenopausal women (letter). *Lancet* **337**: 1414.

Terada S, Uchide K, Suzuki N and Akasofu K (1993) A follicular cyst during tamoxifen therapy in a premenopausal breast cancer woman. *Gynecol Obstet Invest* **35**: 62–64

Van Leeuwen FE, Benraadt J, Coebergh JWW, Kiemeney LALM, Gimbrere CHF, Otter R, Schouten LJ, Damhuis RA, Bontenbal M, Diepenhorst FW, Van den Belt-Dusebout AW and Van Tinteren H (1994) Risk of endometrial cancer after tamoxifen treatment of breast cancer. *Lancet* **343**: 448–452

Whittemore AS, Harris R and Itnyre J (1992) Characteristics relating to ovarian cancer risk: collaborative analysis of 12 US case-control studies. *Am J Epidemiol* **132**: 1184–1203

 *© Cancer Research Campaign 1999*
